# Abortion Is a Right: Perspectives of Family Medicine Physician Residents

**DOI:** 10.7759/cureus.31506

**Published:** 2022-11-14

**Authors:** Anita Vasudevan, Briga Mullin, Reece Fenning, Guille Freschl, Jacqueline Mostow, Hannah Bogen, Aljanee Whitaker, Zach Anderson, Alex Su, Seethim Naicker, Yvonne Chow, Jennifer Tsai, Basilia Oseguera, Justin Chin

**Affiliations:** 1 Family Medicine, Sutter Santa Rosa Regional Hospital, Santa Rosa, USA; 2 Family Medicine, Contra Costa Regional Medical Center, Martinez, USA; 3 Family Medicine, Kaiser Permanente Vallejo Medical Center, Vallejo, USA; 4 Family Medicine, Kaiser Permanente Santa Rosa, Santa Rosa, USA; 5 Family Medicine, Kaiser Permanente San Jose Medical Center, San Jose, USA; 6 Family Medicine, John Muir Health, Walnut Creek, USA; 7 Family Medicine, LifeLong Medical Care, Richmond, USA; 8 Medical Education, Lake Erie College of Osteopathic Medicine, Erie, USA

**Keywords:** abortion 2022, abortion laws and society, abortion ban and restrictions, abortion training, bodily autonomy, healthy equity, health policy and advocacy, racism, structural racism, roe v. wade

## Abstract

Abortion is healthcare. Bodily autonomy is a fundamental human right. As chief resident physicians representing family medicine residency programs in the Greater Bay Area and Central California in the University of California San Francisco Family Medicine Alliance, we share a deep commitment to promoting health equity, advancing social justice, and eliminating health disparities. The 2022 Supreme Court decision in *Dobbs v. Jackson Women's Health Organization* to overturn *Roe v. Wade* contradicts the inherent rights of patients to make their own reproductive healthcare decisions. This is a clarion call for all people to engage in activities to raise awareness and prompt discussion about abortion, reproductive rights, and maternal mortality.

## Editorial

As family medicine resident physicians, we were appalled and outraged by the Supreme Court decision in *Dobbs v. Jackson Women's Health Organization* to overturn *Roe v. Wade* and *Planned Parenthood v. Casey* - in a move that strikes down decades of precedent. As the future of medicine and primary care, we are acutely aware of how measures to protect bodily autonomy are not only just but also lifesaving. We fear a world in which abortion is inaccessible and even criminalized, a world that generations of physicians before us had to navigate. We worry for the patients who will no longer have the right to make the medical decisions that are best for their bodies, their lives, and their families. We worry for our colleagues practicing in states swiftly banning abortion access as they tell their patients that they cannot provide the care they need without the threat of being punished by law. The consequences and ramifications of this decision have been chilling as seen in news media, ethics reviews, and even the potential influence on global healthcare practices [1].

This decision is also an issue of health equity and racism. The most affected populations will be Black, Indigenous, and People of Color who already experience racism, less healthcare access, and higher maternal mortality rates [2,3]. As we work and serve underresourced and marginalized communities, we recognize that the loss of abortion rights will only continue to amplify the inequities in our system [4].

We reaffirm our commitment to our patients and to the fight to protect reproductive rights. We will continue training to become providers who will walk with our patients through any and all decisions they make in pursuit of their health and well-being. We recognize that this commitment to our patients also involves a duty to our colleagues in other parts of the country, and we will actively seek ways to support efforts to expand abortion training for providers who seek to care for patients in areas with less abortion access [5].

In our own clinics, we have often sat with our newly pregnant patients and helped them navigate their questions and emotions. We have held their hands as they chose to terminate pregnancies, created space for their grief as they experienced painful miscarriages for very desired pregnancies, shared in their joy as we deliver their healthy newborns into the world, and cared for both the birthing patient and baby through life-threatening postpartum or neonatal medical complications. It is precisely because of our growing expertise and our respect for human life that we maintain that above all else, we must trust our patients when they decide what is best for their bodies, their lives, and their families.

Our message is clear: *Abortion is healthcare. Bodily autonomy is a fundamental human right.*
